# A remarkable new species of *Pamianthe* (Amaryllidaceae) from the Department of Cauca, Colombia

**DOI:** 10.3897/phytokeys.115.30755

**Published:** 2019-01-17

**Authors:** Alan W. Meerow, Philip A. Silverstone-Sopkin, Alejandro Zuluaga-Tróchez, Jhon A. Sánchez-Taborda

**Affiliations:** 1 USDA-ARS-SHRS, National Germplasm Repository, 13601 Old Cutler Road, Miami, Florida 33158, USA USDA-ARS-SHRS Miami United States of America; 2 Departamento de Biología, Universidad del Valle, Calle 13 # 100-00, Cali, Valle, Colombia Universidad del Valle Cali Colombia; 3 Fundación Ecohábitats, Calle 78N # 19-157, Popayán, Cauca, Colombia Fundación Ecohábitats Popayán Colombia

**Keywords:** Amaryllidaceae, biodiversity, Cauca, Clinantheae, Colombia, *Pamianthe*, Andes

## Abstract

A new saxicolous species of Amaryllidaceae tentatively assigned to the tribe Clinantheae, *Pamiantheecollis* Silverst., Meerow & Sánchez-Taborda, is described from the western slope of the Cordillera Occidental in the department of Cauca, Colombia. The new species differs from the two hitherto known species of *Pamianthe* in its yellow flowers and in its nearly obsolete perianth tube. The near loss of the perianth tube may be correlated with a change in pollinator. The new species lacks a bulb; it produces a large number of winged seeds that are wind-dispersed. A key to the species of *Pamianthe* is provided. This is the first record of the genus *Pamianthe* for Colombia. The phylogenetic position of the genus *Pamianthe* is discussed.

## Abstract (es)

Se describe una nueva especie de Amaryllidaceae tentativamente perteneciente a la tribu Clinantheae, *Pamiantheecollis* Silverst., Meerow & Sánchez-Taborda, procedente de la vertiente occidental de la cordillera Occidental en el departamento del Cauca, Colombia. La nueva especie difiere de las dos especies conocidas de *Pamianthe* por su perianto amarillo que tiene un tubo casi ausente. La reducción del tubo del perianto probablemente tiene correlación con un cambio en el polinizador. La nueva especie carece de un bulbo, y produce numerosas semillas aladas que se dispersan por el viento. Se provee una clave a las especies de *Pamianthe.* Este es el primer registro del género *Pamianthe* para Colombia. Se discute la posición filogenética del género *Pamianthe*.

## Introduction

Amaryllidaceae J. St.-Hil. is a cosmopolitan family represented in Colombia by nine native genera and 26 native species, including a monotypic endemic genus, *Plagiolirion* Baker (Meerow and Silverstone-Sopkin 1995). Some of the Colombian species have restricted ranges and are in danger of extinction or may already be extinct (Silverstone-Sopkin 2011). Recent field work in the Cordillera Occidental of the Andes, in the department of Cauca, has resulted in the discovery of a new species of Amaryllidaceae that also seems to be narrowly distributed. Vegetative and floral morphology and nrDNA ITS sequences indicate that this species represents a novelty in the genus *Pamianthe* Stapf.

Stapf (1933a, 1933b) published the genus *Pamianthe* in honor of Major Albert Pam, who cultivated bulbs in England that he received from Peru in 1928. There are five published species names that have been assigned to this genus: *P.andreana* (Baker) Stapf, *P.cardenasii* Traub, *P.parviflora* Meerow, *P.peruviana* Stapf, and *P.quitoensis* (Herb.) Stapf. *Pamianthequitoensis* was transferred to the genus *Leptochiton* Sealy, as *L.quitoensis* (Herb.) Sealy, and *P.andreana* is considered a synonym of this species. *Pamianthecardenasii* has been placed in the synonymy of *P.peruviana* (Meerow 1984). Thus, the genus *Pamianthe*, as previously recognized, includes only two species, *P.parviflora*, known only from Ecuador (Meerow 1984), and *P.peruviana* (the type species), known from Perú and Bolivia. The new species described in this paper is the third species of the genus and the first record from Colombia. It is also the first species of the tribe Clinantheae, to which *Pamianthe* has been assigned (Meerow et al. 2000; Leiva and Meerow 2016), discovered north of Ecuador.

## Methods

Photographs of the flower in alcohol and seeds of *Pamiantheecollis* were taken with a Nikon model DS-Ri1U3 digital camera, using a Nikon model SMZ-1500 stereo dissecting microscope at the Laboratorio de Imágenes del Postgrado en Ciencias-Biología de la Universidad del Valle; floral and seed measurements were made with NIS Elements Br, version 4.20 software.

DNA extraction, amplification and sequencing protocols were as described in Meerow et al. (2000, 2006). The ITS sequence of *P.ecollis* was aligned with a previous ITS alignment of the tribe Clinantheae (Meerow et al. 2000; Meerow 2010) using the program MAFFT (Katoh and Standley 2013). A branch and bound parsimony analysis was run using PAUP v. 4.10 (Swofford 2002), followed by generation of Jackknife support percentages. The ITS sequence of *P.ecollis* is deposited in GenBank (Genbank Acc. MH979036).

## Results

### Taxonomic treatment

#### Pamianthe ecollis

Taxon classificationPlantaeAsparagalesAmaryllidaceae

Silverst., Meerow & Sánchez-Taborda sp. nov.

urn:lsid:ipni.org:names:77193890-1

Figs 1 , 2

##### Diagnosis.

This species differs from both *Pamiantheparviflora* Meerow and *P.peruviana* Stapf in having a yellow perianth and staminal cup (versus white) and in nearly lacking a perianth tube. Additionally, it differs from *P.parviflora* in having shorter pedicels, a longer ovary, and more numerous ovules, and from *P.peruviana* in having much longer pedicels, more flowers per umbel, much shorter tepals, a shorter staminal cup that is not exserted from the perianth, and a smaller fruit.

##### Type.

COLOMBIA. Cauca: Municipio Argelia, road between Nuevo Horizonte and La Montaña, north of the Serranía El Pinche, Cordillera Occidental, western slope (Fig. 3), 2839 m, 4 Feb 2018, *J. A. Sánchez-Taborda 2870* (holotype: CUVC 67719!, 67720!, mounted on two sheets; isotype: CAUP). GPS coordinates are withheld to discourage poaching; they are available to bonafide researchers upon request.

##### Description.

**Figure 1.**
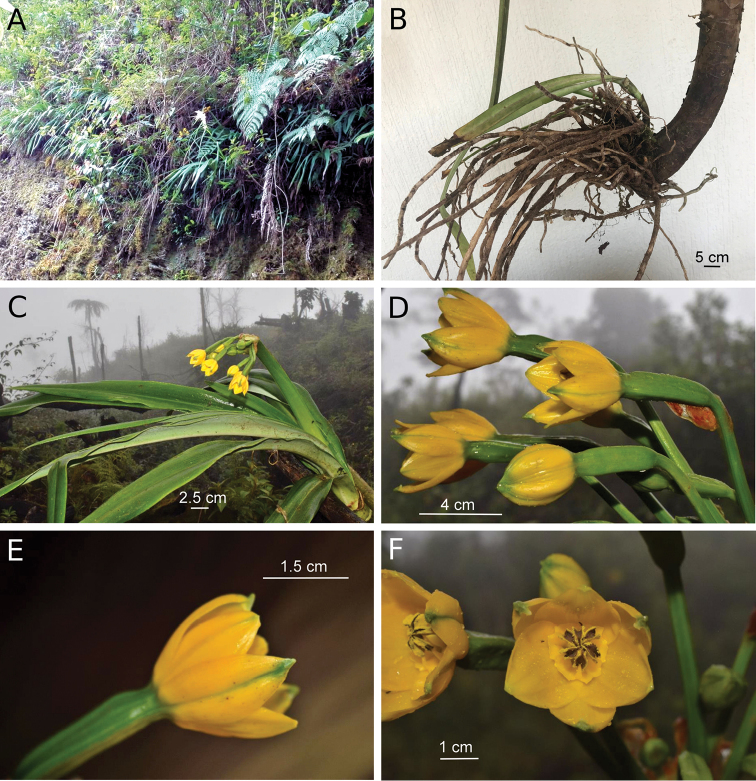
*Pamiantheecollis*. **A***Pamiantheecollis* growing in its native habitat, on a steep, rocky bank **B** Base of plant **C** Habit **D** Inflorescence **E** Flower, lateral view **F** Flower, front view **A** photo by Fredy Gómez-Ortiz **B** photo by Laura Clavijo **C–F** type collection, photographs taken in the field by Jhon A. Sánchez-Taborda.

**Figure 2.**
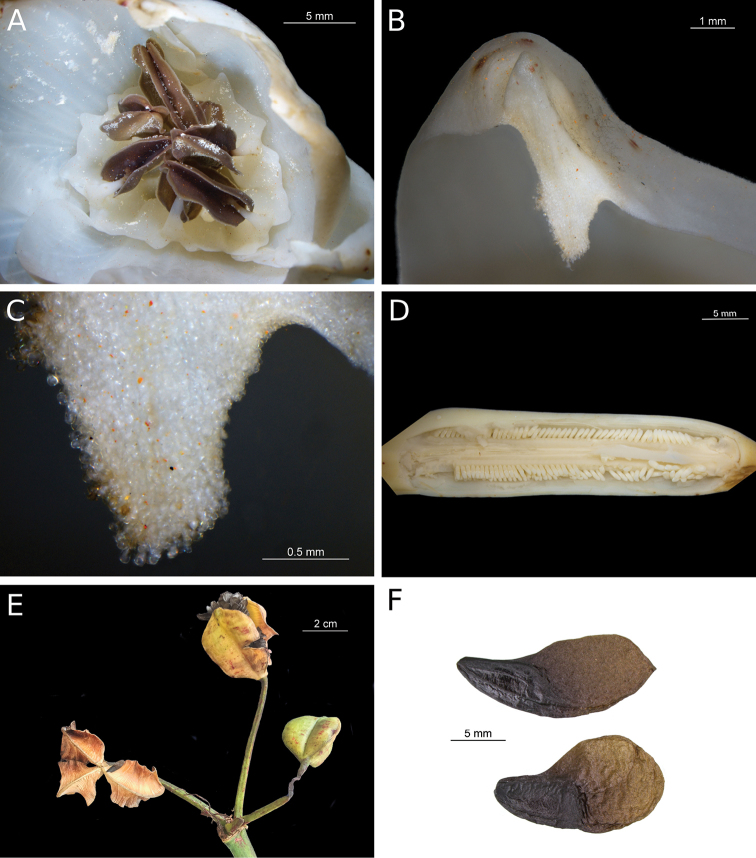
*Pamiantheecollis*. **A** Androecium, with staminal cup **B** Tip of outer tepal, showing apex and adaxial protuberance **C** Adaxial protuberance, showing glandular papillae **D** Opened ovary with ovules (ovules in two locules are visible) **E** Infructescence of living plant **F** Seeds, showing variation in shape **A–D, F** photographs by Juan Felipe Ortega-Giraldo, Laboratorio de Imágenes del Postgrado en Ciencias-Biología, Universidad del Valle, Cali, Colombia **E** photo by Laura Clavijo.

Terrestrial saxicolous herbs (Fig. 1A); bulb absent, roots emerge from base of pseudostem, and are thick, possibly with a velamen layer (Fig. 1B). Leaves (Fig. 1C) sessile, attached alternately to an elongate pseudostem; lamina lorate, 82.7–104.5 × 5.5–6.3 cm, margin entire, glabrous, narrowing distally (but not acuminate), apex acute, with a conspicuous midrib. Scape cylindrical, 45–46 cm long; intact bracts not seen (bracts withered and damaged in dried specimens); inflorescence pseudoumbellate, flowers oriented at right angles from apex of pedicels. Flowers (Fig. 1D–F) 9–10, of which 3–4 are at anthesis simultaneously; pedicels in flowers at anthesis 7–9 cm long; perianth tube nearly obsolete (ca. 1.8 mm long); limb crateriform, ca. 3.3 cm in diam; tepals 6, yellow, glabrous; outer tepals with green tips and very narrow green abaxial mid-longitudinal stripe, valvate, elliptical, ca. 3.2 × 1.4–1.5 cm, apiculate, apex thickened, ca. 2.3 mm long, with salient adaxial apiculum (Fig. 2B) ca. 1.3 × 1.4 mm, which is densely glandular-papillate (Fig. 2C); inner tepals imbricate at base, ovate, broader than outer tepals, ca. 2.8 × 2.1 cm, apex rounded, thickened and papillate on adaxial surface, but not apiculate and lacking adaxial protuberance. Stamens 6, basally connate into immaculate yellow staminal cup attached to the adaxial base of inner tepals (Fig. 2A), ca. 5 mm long (measured from base to tip of tooth), not exserted, with 2 deltoid to rounded teeth between each 2 free filaments; free filaments yellow, ca. 5 mm long, attached to border of staminal cup, included, strongly incurved; anthers grouped in center of flower (but not connivent), brown with yellow borders, ca. 7.1 mm long, linear, dorsifixed, versatile, longitudinally dehiscent; pollen yellow. Style (in the only flower preserved in ethanol) apparently immature (flower protandrous), curved, ca. 10 mm long, included (hidden below the grouped anthers), stigma 3-lobed, lobes papillate; ovary green, 3-angled, oblong, ca. 40 × 9 mm, 3-loculed, placentation axile, ovules oblong, ca. 1.6 × 0.5 mm, ca. 100 per locule (Fig. 2D), biseriate, ovules of each row alternating with those of the other row. Fruit (Fig. 2E): unopened fruit not available for measurement; dehiscent fruit 3-valved, valves broad-elliptic to obovate, base obtuse, apex short-beaked, dry, smooth, glabrous, ca. 38 × 29 mm. Seeds (Fig. 2E, F) as many as 233 in one capsule, alate, glabrous, seed body dark brown, wing light brown, flat, thin, membranous, shape of entire seed (including wing) narrowly to broadly falcate, (12–) 15–18 × 5–9 mm.

##### Distribution and ecology.

**Figure 3.**
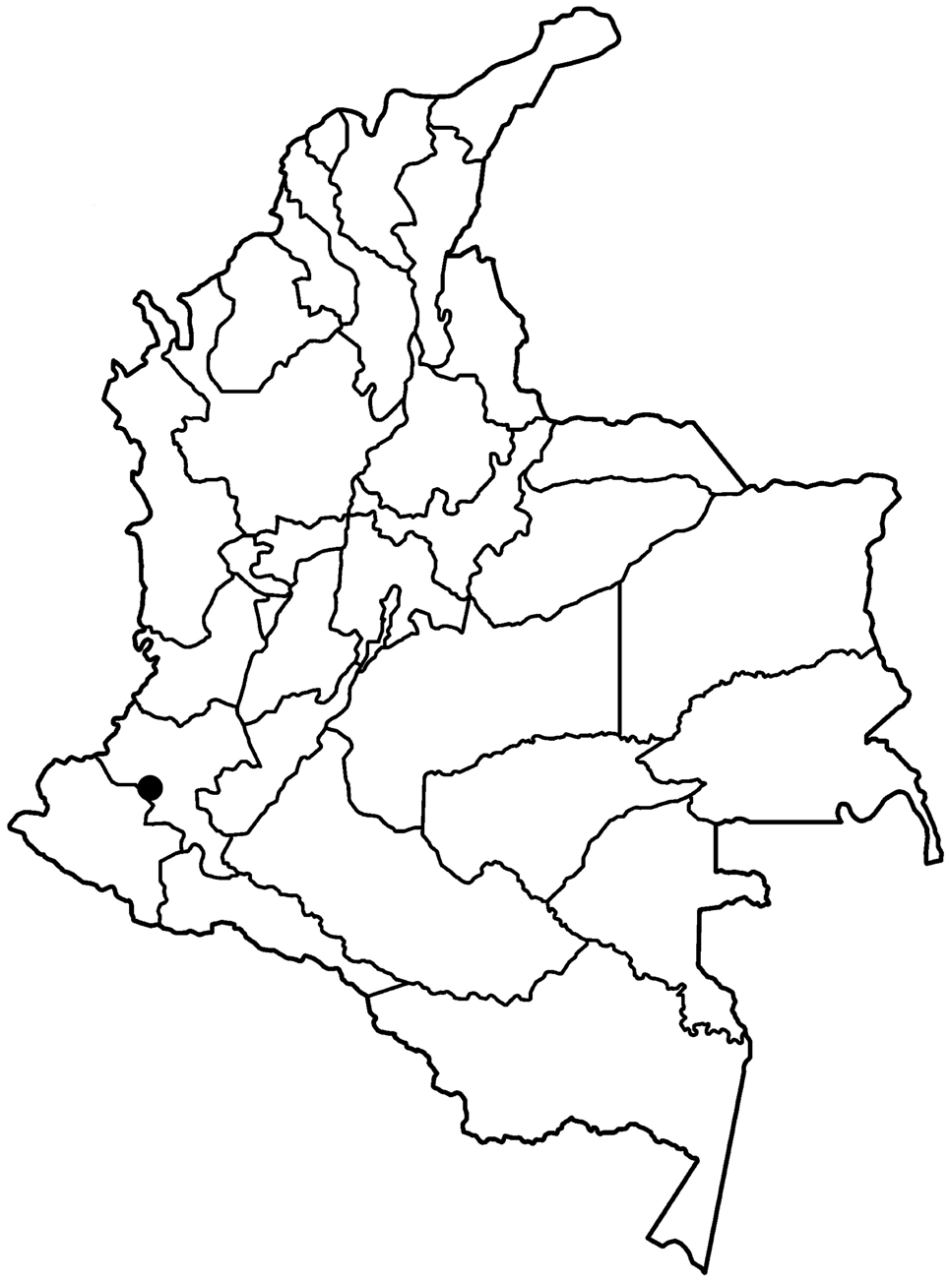
Map of Colombia showing the distribution of *Pamiantheecollis* (black circle).

*Pamiantheecollis* is known only from the type locality (Fig. 3). The general habitat is cloud forest. The forest at this site includes the following genera: trees: *Clusia* L., *Hedyosmum* Sw., *Ocotea* Aubl.; shrubs: *Miconia* Ruiz & Pav., *Palicourea* Aubl.; herbs: *Anthurium* Schott, *Besleria* L., *Kohleria* Regel, *Peperomia*Ruiz & Pav., and *Sphaeradenia* Harling. Epiphytes were predominantly bromeliads and orchids. The new species is common at this site (Fredy Gómez-Ortiz pers. com.). However, this species does not grow within closed forest. The seeds of *P.ecollis*, which are adapted for anemochory, and a photograph of the population at the type locality (Fig. 1A), indicate that this species inhabits open areas on steep banks near creeks. Plants from the type collection were growing near a waterfall. Plants from a later collection, from which herbarium specimens were not prepared, were growing on an apparently disturbed, open slope on rocky substrate. The roots of the plants are superficial, immersed in a thick layer of moss, and grasp the surface of the rock. Thus, this species is a lithophyte.

##### Phenology.

Plants were collected in flower in February and in fruit in August.

##### Etymology.

The specific epithet is from Latin, *e* (without), *collum* (neck), adjectival *collis*, referring to the almost absent perianth tube of this species.

##### Preliminary conservation status.

Since nothing is known of the distribution of this species apart from the type locality, it is best to place it in the category Data Deficient (IUCN 2012, 2017).

## Discussion

**Figure 4.**
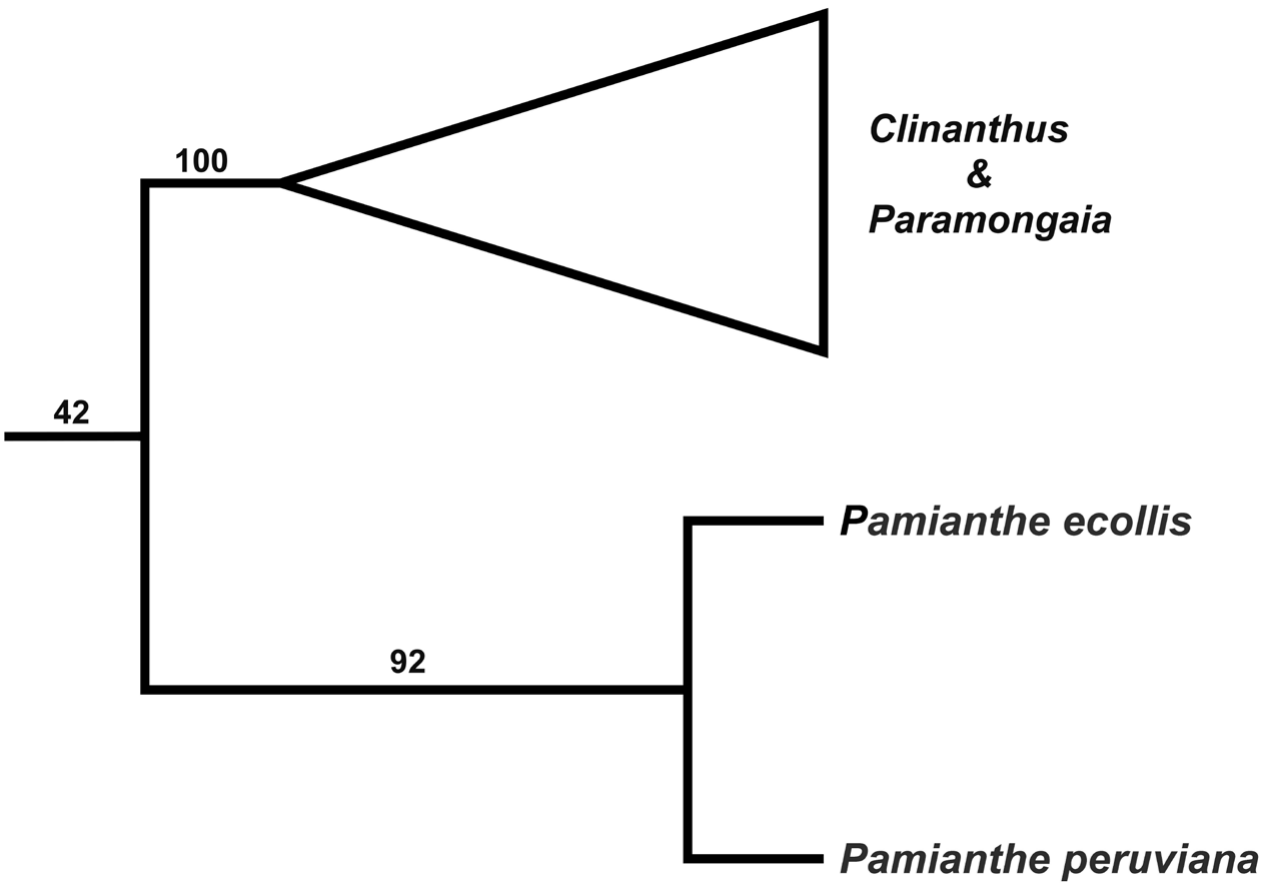
Strict branch and bound parsimony consensus tree of the Clinantheae, based on ITS sequences, with jackknife support values.

A strict consensus tree cladogram (Fig. 4) based on ITS sequences of the tribe Clinantheae places the new species of *Pamianthe* as sister to *P.peruviana* with 92% jackknife support, in a subclade that is sister to a second subclade comprising *Clinanthus* Herb. and *Paramongaia* Velarde. However, with ITS there is no support for *Pamianthe* as part of Clinantheae (jackknife support = 42%; Fig. 4). Preliminary super matrix trees from sequence capture with anchored bait enrichment (Meerow, unpublished data) suggest that *Pamianthe* is in fact sister to the tribes Clinantheae, Eucharideae, and Hymenocallideae, rather than the first branch in Clinantheae.

*Pamiantheecollis* resembles the two other species of *Pamianthe* in its staminal cup morphology, with the free portion of the staminal filaments attached to the rim of the cup (not below the rim), two lobes or teeth between each two staminal filaments, and the staminal filaments strongly curved inward, as well as numerous, biseriate, winged, wind-dispersed seeds. Leaf width and the conspicuous midvein are similar to that of *P.peruviana*. It differs from both of the two hitherto known species in having a yellow perianth and staminal cup (versus white in the other two species) and in its nearly obsolete perianth tube. Moreover, *P.parviflora* has a shorter ovary (10 mm versus 40 mm in *P.ecollis*) and fewer ovules per locule (about 20 versus about 100 in *P.ecollis*). *Pamiantheperuviana* additionally differs in having fewer flowers (2–4, usually 2, versus 9–10 in *P.ecollis*), shorter pedicels (1.5–3 cm long versus 7–9 cm long in *P.ecollis*), free tepals much longer (outer tepals 10–12 cm long, inner tepals 9–11 cm long, versus 3.2 and 2.8 cm long in *P.ecollis*), staminal cup 8 cm long and long-exserted (versus ca. 0.5 cm long and included in *P.ecollis*), and larger fruit (8 cm long, 5 cm wide, versus 3.8 cm long, 2.9 cm wide in *P.ecollis*).

The elongate (12–25 cm long) perianth tube in *P.peruviana*, which contains three nectar-bearing internal channels (Traub 1972), may be correlated with pollination by sphingid moths. The nearly obsolete perianth tube of *P.ecollis* may be associated with a change in pollinators; in a tubeless perianth, nectar would be available to short-tongued insects, such as bees. No flower visitors have been observed.

The glandular papillae (Fig. 2C) on the adaxial protuberance of the outer tepals apparently have a secretory function. They probably play a role in pollinator attraction; they may produce a substance that is gathered by insect visitors, or they may function as osmophores. Possible osmophores have been reported in the Chilean allioid amaryllid *Gilliesia* Lindl. (Rudall et al. 2002). The flat, alate seeds are most likely wind-dispersed, suggesting that these plants inhabit open areas within the cloud forest vegetation; seeds of Amaryllidaceae of closed lowland tropical forest, such as *Eucharis* Planch. & Lind., are relatively few per locule, subglobose, and wingless, and probably are bird-dispersed, and in one case possibly water-dispersed (Silverstone-Sopkin 2011).

The Clinantheae, which is sister to the tribe Hymenocallideae (Meerow et al. 2000), was not previously known to extend to Colombia. We hypothesize that the three rare species of *Pamianthe* may represent the remnants of a once more broadly distributed epiphytic and lithophytic lineage in the tribe that were isolated as the Andes rose to their present position, and moist forests contracted on the western slopes. We are confident that rigorous analysis of our next generation sequence data will successfully resolve the current ambiguous phylogenetic position of the genus.

### Key to the species of the genus *Pamianthe*

**Table:** 

1	Perianth and staminal cup yellow, perianth tube nearly obsolete	***Pamiantheecollis* Silverst., Meerow & Sánchez-Taborda**
–	Perianth and staminal cup white, perianth with a well-developed tube	**2**
2	Pedicels 5–6 cm long; perianth tube less than 2 cm long; outer tepals less than 3 cm long; staminal cup less than 2 cm long	***Pamiantheparviflora* Meerow**
–	Pedicels 1.5–3 cm long; perianth tube more than 11 cm long; outer tepals more than 8 cm long; staminal cup more than 7 cm long	***Pamiantheperuviana* Stapf**

## Supplementary Material

XML Treatment for Pamianthe ecollis
